# Elevation of circular RNA circ_0005230 facilitates cell growth and metastasis via sponging miR-1238 and miR-1299 in cholangiocarcinoma

**DOI:** 10.18632/aging.101872

**Published:** 2019-04-04

**Authors:** Yi Xu, Yue Yao, Yueping Liu, Zhidong Wang, Zhanliang Hu, Zhilei Su, Chunlong Li, Hao Wang, Xingming Jiang, Pengcheng Kang, Dianjun Sun, Xiangyu Zhong, Yunfu Cui

**Affiliations:** 1 Department of Hepatopancreatobiliary Surgery, the Second Affiliated Hospital of Harbin Medical University, Harbin 150086, China; 2 Department of Endocrinology and Metabolism, the Second Affiliated Hospital of Harbin Medical University, Harbin 150086, China; 3 The Key Laboratory of Myocardial Ischemia, Harbin Medical University, Ministry of Education, Harbin 150086, China; 4 Center for Endemic Disease Control, Chinese Center for Disease Control and Prevention, Harbin Medical University, Harbin 150086, China; * Equal contribution

**Keywords:** cholangiocarcinoma, circular RNA, circ_0005230, miR-1238, miR-1299

## Abstract

Cholangiocarcinoma (CCA) is a highly malignant carcinoma with high mortality rate worldwide. Emerging evidence indicates that aberrantly expressed circular RNAs (circRNAs) functions crucial roles in tumor progression. In this work, we focused on a novel circRNA, circ_0005230, in carcinogenesis and development of CCA. Circ_0005230 levels in CCA specimens and cells were measured by qRT-PCR. The clinical implication of circ_0005230 was analyzed by fisher’s exact test. Gain/loss of-function assays were conducted to reveal the effects of circ_0005230 on the cell proliferation, apoptosis, migration and invasion of CCA cells. Xenograft and lung metastatic models were constructed to confirm the *in vitro* data. Dual luciferase reporter and rescue assays were carried out to illuminate the mechanism behind the regulatory actions. As data showed, circ_0005230 was elevated in tumors and CCA cells. Its expression in tumor samples was related to clinical severity. Functionally, circ_0005230 significantly facilitated cell growth, clone-forming ability and metastatic properties and inhibit cell apoptosis in CCA cells. The *in vivo* study further validated the *in vitro* results. However, knockdown of circ_0005230 did not affect normal biliary epithelial (HIBEC) cell growth and apoptosis. For the mechanism investigation, circ_0005230 could directly sponge miR-1238 and miR-1299 to exert its oncogenic functions. Overall, this work showed that circ_0005230 might act as an effective therapeutic target for CCA.

## Introduction

Cholangiocarcinoma (CCA) is a digestive carcinoma with a high mortality rate across the world [1,2]. It is originated from the neoplastic transformation of biliary epithelial cells with high malignancy. Based on public data, the 5-year overall survival of this devastating disease is less than 40% [3]. Due to the limit approaches for CCA early detection, many patients lost their chance for radical surgery. Although great advancements in surgical technique and neoadjuvant chemoradiotherapy have been achieved, the prognosis of CCA patients remains unfavorable [4]. Therefore, it is urgent to identify the underlying mechanisms in regulating CCA initiation and progression.

Studies have documented that more than 90% of the human genome is transcribed into non-coding RNAs (ncRNAs), indicating that ncRNAs might play crucial roles in cellular physiology and pathology processes [5,6]. Circular RNAs (circRNAs) is a class of ncRNAs which has limited protein coding potential and formed by a circular structure [7,8]. Accumulating evidence documented that circRNAs are closely involved in carcinogenesis and cancer progression [9,10]. Mechanistically, the studies demonstrated that circRNAs could sponge miRNAs or function as molecular scaffolds to guide RNA binding proteins (RBPs) [11]. For example, a recent study indicated that decreased circRNA_100269 in gastric cancer could suppress cancer cell growth by targeting miR-630 [12]. Nevertheless, the study relevant to circRNAs and CCA is still on the way. Previously, we identified circ_0001649 was downregulated in CCA and ectopic expression of circ_0001649 exerts tumor suppressive role in cell progression [13]. CircRNAs exploration might provide the researches a novel insight into the pathogenesis of this fatal disease.

**Figure 1:**
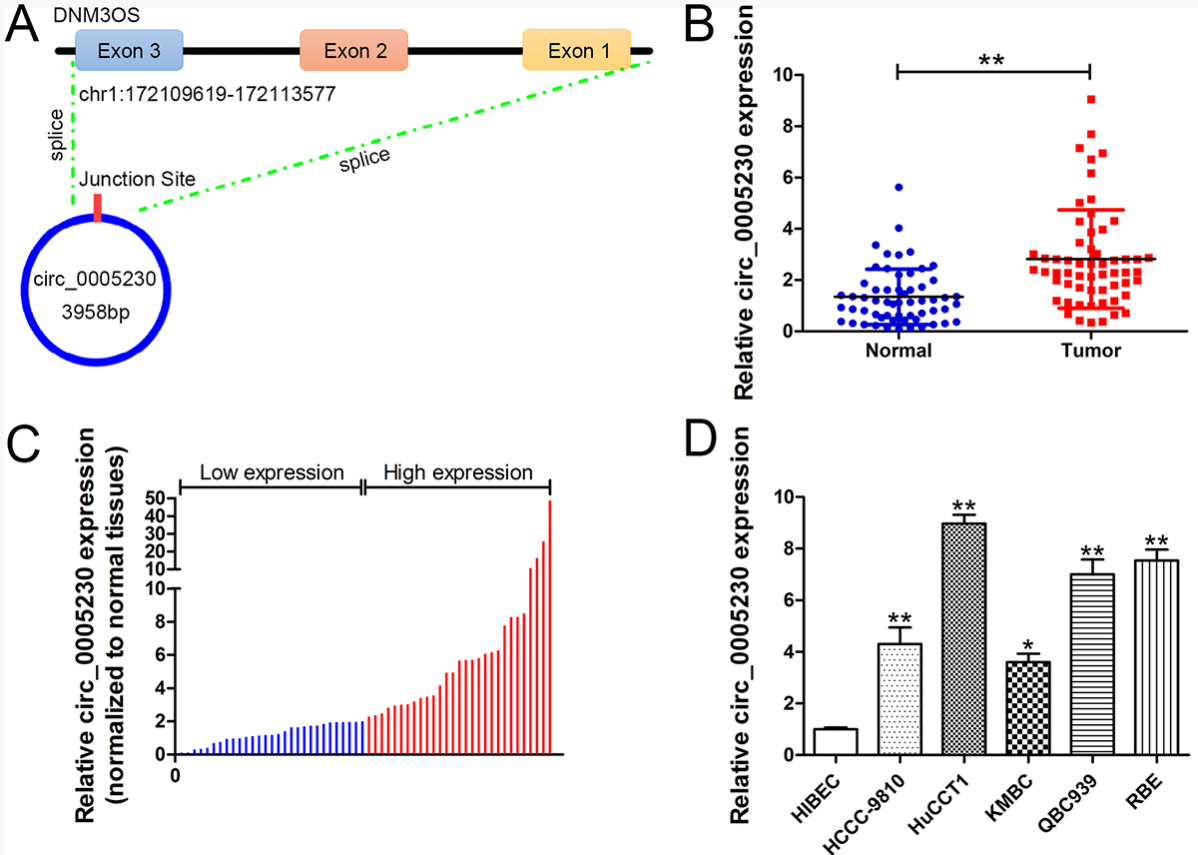
**Relative expression of circ_0005230 in CCA tissues and cell lines and its clinical significance.** (**A**) A schematic diagram of the genomic location and splicing pattern of circ_0005230. (**B**) Relative expression of circ_0005230 in CCA tissue samples and their paired non-cancerous tissue samples measured by qRT-PCR. (**C**) The patients were classified into two groups according to circ_0005230 expression. (**D**) Relative expression of circ_0005230 in CCA cell lines and normal cell line measured by qRT-PCR. *p<0.05, **p<0.01.

Previously, we identified circ_0005230 was elevated in breast cancer tissue samples and was linked to breast cancer patients’ unfavorable prognosis. Furthermore, the biological functions of circ_0005230 was partially attributed to its modulation on miR-618/CBX8 signaling [14]. Circ_0005230 is located at chr1:172109619-172113577 and is spliced from DNM3OS (DNM3 opposite strand/antisense RNA) gene (Fig. 1A). In this work, we further determined circ_0005230 expression in CCA tissue samples and cell lines. Additionally, loss/gain of-function experiments were conducted to investigate cell growth, apoptosis, migration, and invasion potential affected by circ_0005230. Moreover, *in vivo* study was performed to validate the *in vitro* data. Importantly, the underlying mechanism was explored by dual luciferase reporter assays. Collectively, this work may help to form a novel effective therapeutics target for treating this devastating disease.

## RESULTS

### Circ_0005230 expression is enhanced in CCA tissues and cells and correlates with patients’ clinical severity

The data from qRT-PCR uncovered that circ_0005230 expression was evidently enhanced in CCA specimens compared with the healthy tissues (2.10-fold, Fig. 1B). Therefore, fifty eight recruited CCA patients could be divided into high (>2.10) and low (<2.10) expression groups (Fig. 1C). Furthermore, similar results were identified in CCA cells relative to the normal biliary epithelial cells (HIBEC, Fig. 1D). After validating the elevation of circ_0005230 in CCA specimens and cell lines, its clinical implication was investigated. As analysis indicated, circ_0005230 expression is related to larger tumor size (*p*=0.028), positive lymph node invasion (*p*=0.003), and advanced TNM stages (*p*=0.020) for CCA patients. The other features were not linked to circ_0005230 expression (gender, age, tumor site, and differentiation grade, Table 1).

**Table 1:** Relationship between circ_0005230 expression and clinicopathologic features of CCA patients.

Clinicopathologic	patients	circ_0005230 expression		*p*-value
features		High	Low	
Gender				
Male	27	15(25.86%)	12(20.69%)	0.599
Female	31	14(24.14%)	17(29.31%)	
Age				
<60	30	17(29.31%)	13(22.41%)	0.431
≥60	28	12(20.69%)	16(27.59%)	
Tumor site				
Intrahepatic	15	6(10.34%)	9(15.52%)	0.550
Extrahepatic	43	23(39.66%)	20(34.48%)	
Tumor size				
<3cm	37	14(24.14%)	23(39.66%)	**0.028**
≥3cm	21	15(25.86%)	6(10.34%)	
Lymph node invasion				
Present	36	24(41.38%)	12(20.69%)	**0.003**
Absent	22	5(8.62%)	17(29.31%)	
TNM stage				
I-II	17	4(6.90%)	13(22.41%)	**0.020**
III-IV	41	25(43.10%)	16(27.59%)	
Differentiation grade				
Well/moderately	20	8(13.79%)	12(20.69%)	0.408
Poorly/undifferentiated	38	21(36.21%)	17(29.31%)	

### Circ_0005230 facilitates HuCCT1 and KMBC cell progression *in vitro*

**Figure 2:**
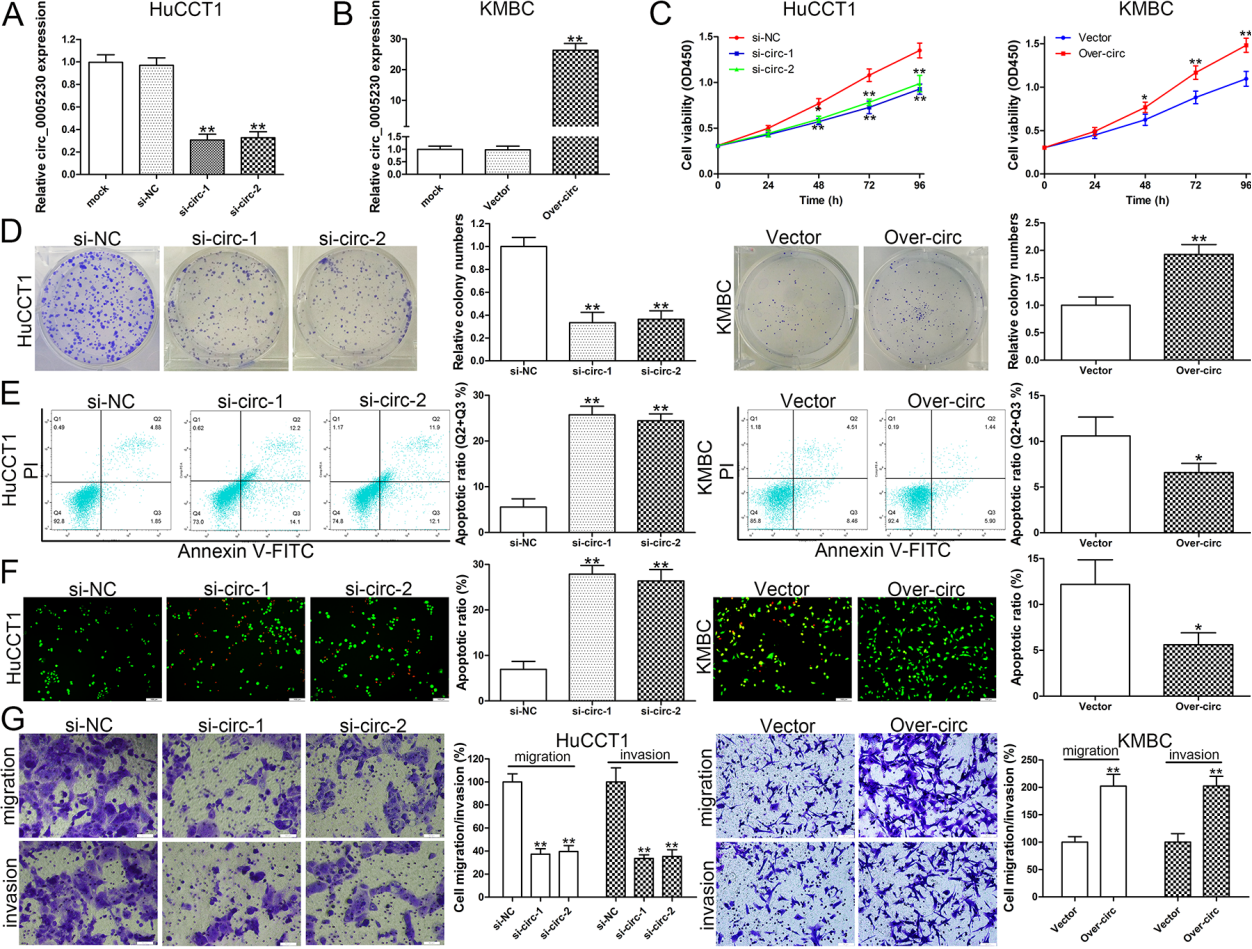
**Circ_0005230 regulates cell growth, apoptosis, migration and invasion.** (**A**) Circ_0005230 expression was detected after transfection in HuCCT1 cells by qRT-PCR. (**B**) Circ_0005230 expression was detected after transfection in KMBC cells by qRT-PCR. (**C**) CCK-8 assays were used to detect cell viability of HuCCT1 and KMBC cells after transfection. (**D**) Colony formation assays were used to detect the clone ability of HuCCT1 and KMBC cells after transfection. (**E**) Flow cytometric analysis were used to detect cell apoptosis of HuCCT1 and KMBC cells after transfection. (**F**) AO/EB staining was used to detect cell apoptosis of HuCCT1 and KMBC cells after transfection. (**G**) Transwell assays were used to detect cell migration and invasion capacities of HuCCT1 and KMBC cells after transfection. *p<0.05, **p<0.01.

SiRNAs were used for transfection to silence circ_0005230 expression in HuCCT1 cells, (Fig. 2A). In addition, circ_0005230 was ectopically expressed in KMBC cells for the reason that it has the lowest level of circ_0005230 (Fig. 2B). Cell growth was evaluated by CCK-8 and clonogenic experiments and the data revealed that cell proliferation was attenuated in the si-circ-1/-2 groups relative to the negative control group. After circ_0005230 overexpression, cell viability and clone forming ability were both enhanced (Fig. 2C and D). We next investigated the effect of circ_0005230 on CCA cell apoptosis by flow cytometry and AO/EB staining assays. Apoptotic cells were measured at 48h post-transfection. Compared to si-NC group, cell apoptotic rate was elevated in si-circ-1 and si-circ-2 groups. On the contrary, cell apoptosis was inhibited after circ_0005230 upregulated in KMBC cells (Fig. 2E). Similar results were found in AO/EB double fluorescence staining assay (Fig. 2F). Cell migratory and invasive assays confirmed that cell metastatic ability was decreased after circ_0005230 knockdown in HuCCT1 cells. Whereas, in comparison with empty vector group, the cells with elevated circ_0005230 had a stronger migratory and invasive capacities (Fig. 2G).

### Knockdown of circ_0005230 expression does not affect HIBEC cell growth and apoptosis

**Figure 3:**
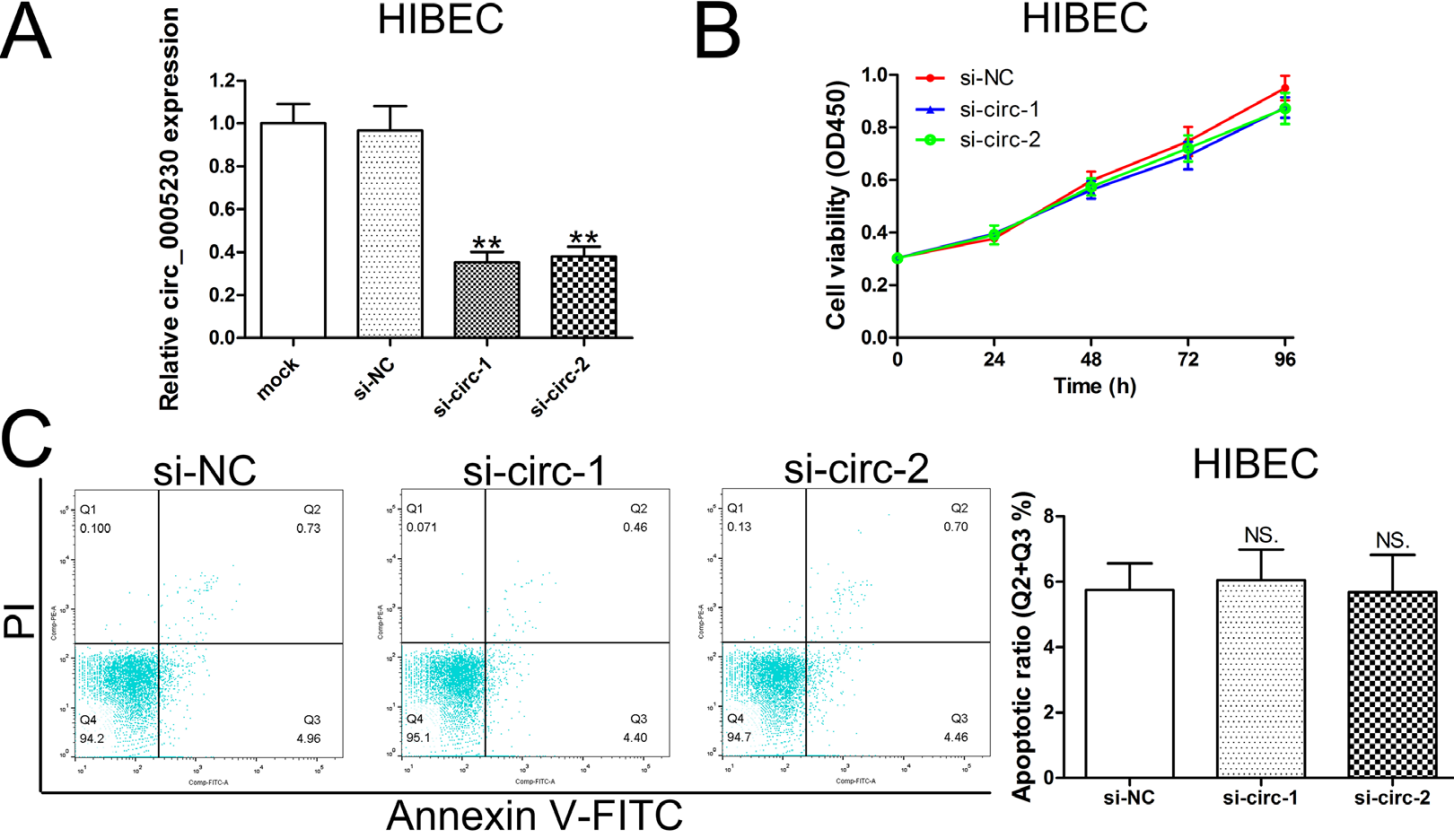
**Decreased expression of circ_0005230 does not affect HIBEC cell proliferation and apoptosis.** (**A**) HIBEC cells were transfected with siRNAs and siRNA-depletion efficiency was detected by qRT-PCR. (**B**) The proliferation of HIBEC cells after transfection was detected by CCK-8 assays. (**C**) The apoptosis of HIBEC cells after transfection was detected by flow cytometry.

In order to clear whether silencing of circ_0005230 has side effect on normal biliary epithelial cells, we silenced circ_0005230 expression in HIBEC cells. As Fig. 3A showed, both of the two siRNAs had effective knockdown efficiencies. However, the cell viability curves based on CCK-8 assay indicated that the growth of HIBEC cells was not altered after circ_0005230 silenced (Fig. 3B). Additionally, apoptotic rate was not statistically significance affected in different transfected groups (Fig. 3C).

### Circ_0005230 directly sponges miR-1238 and miR-1299 in CCA cells

**Figure 4:**
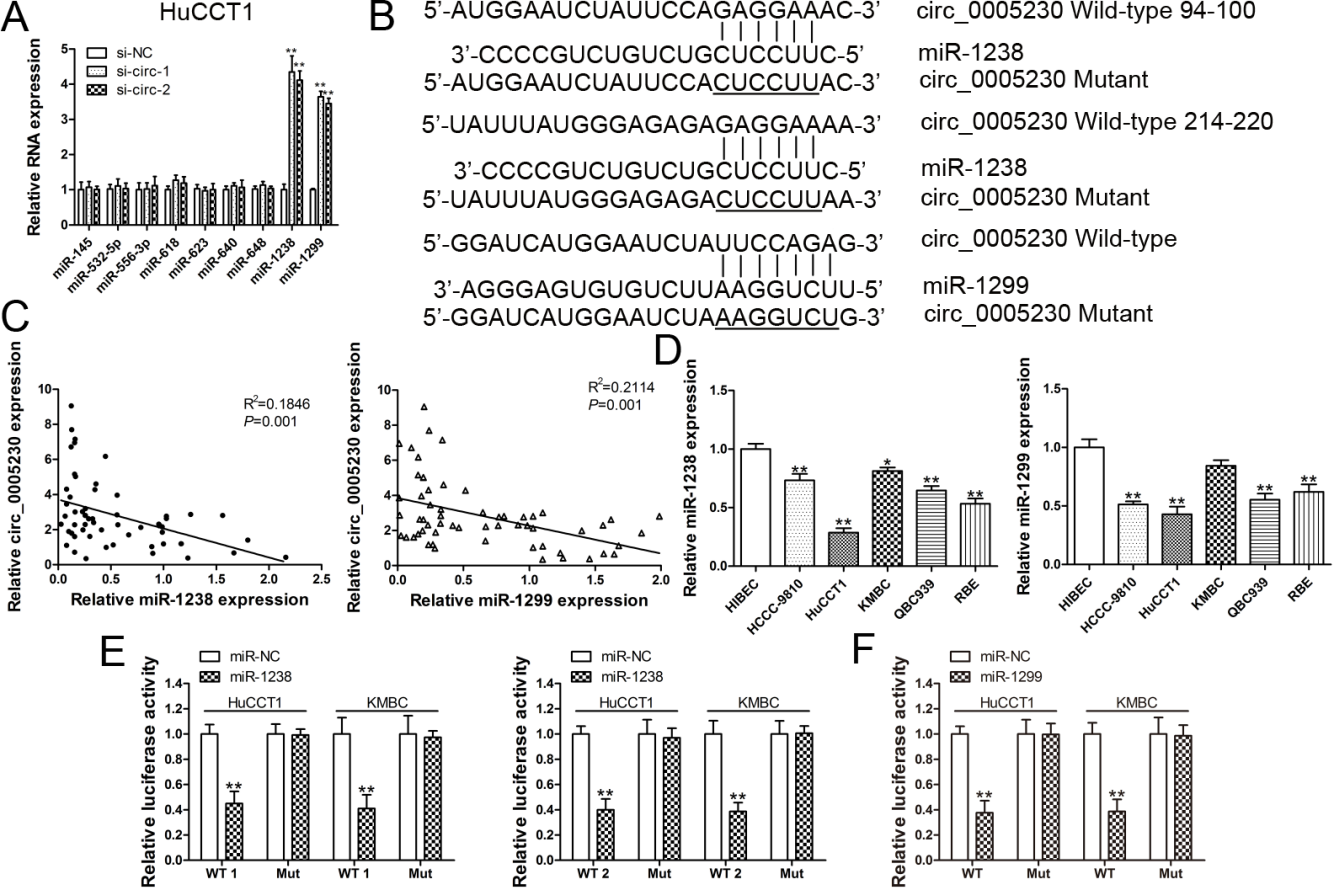
**Circ_0005230 is a miRNA sponge for miR-1238 and miR-1299.** (**A**) MiRNAs expression was detected after silencing of circ_0005230 in HuCCT1 cells. (**B**) Diagrammatic sketch of the binding sites for miR-1238 and miR-1299 in circ_0005230. (**C**) Correlation analysis of circ_0005230 and miR-1238/miR-1299 in CCA patients’ tissues. (**D**) qRT-PCR was performed to detect the expression of miR-1238/miR-1299 CCA cells and HIBEC cells. (**E, F**) Luciferase reporter assay showed that ectopic expression of miR-1238 and miR-1299 suppressed the activity of circ_0005230-WT in HuCCT1 and KMBC cells. *p<0.05, **p<0.01.

Various reports illustrated that circRNAs participates in post-transcriptional regulation by sponging several miRNAs [15,16]. To further evaluate the mechanisms of circ_0005230 exert in CCA, bioinformatics analysis was used to predict miRNAs that may be sponged by circ_0005230. Among all the predicted miRNAs, only miR-1238 and miR-1299 could be regulated by si-circ-1 and si-circ-2. The other recruited miRNAs were not altered statistically significant (Fig. 4A). The binding sites of miR-1238 and miR-1299 within circ_0005230 were shown in Fig. 4B. There are two potential binding sites of miR-1238 within circ_0005230 (94-100 and 214-220). Afterwards, the relative expression of miR-1238 and miR-1299 in CCA cancerous tissues was detected. Correlation analysis documented that either miR-1238 or miR-1299 was negatively correlated with circ_0005230 level in CCA tissue samples (Fig. 4C). For the enrolled CCA and HIBEC cells, we found miR-1238 and miR-1299 were both downregulated in CCA cell lines (Fig. 4D). Finally, we conducted luciferase reporter assay to confirm the binding capacity between miR-1238/1299 and circ_0005230. As Fig. 4E demonstrated, both of the binding sites of miR-1238 on circ_0005230 were functional. In addition, the luciferase activities of HuCCT1 and KMBC cells transfected with miR-1299 and circ_0005230 WT were decreased compared with the scramble control group (Fig. 4F).

### Circ_0005230 facilitates cell progression by sponging miR-1238 and miR-1299

**Figure 5:**
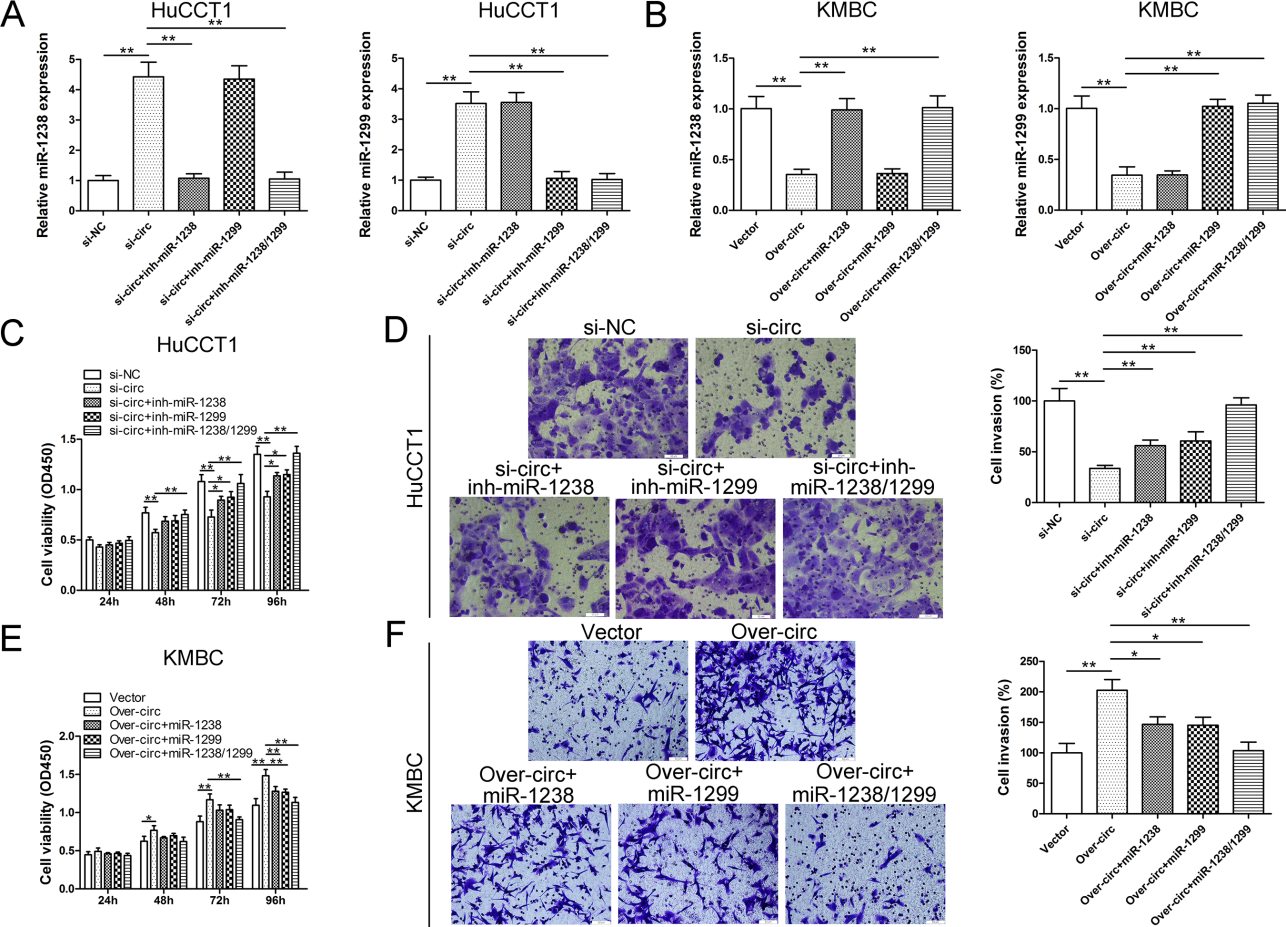
**The oncogenic role of circ_0005230 is partly dependent on its regulation on miR-1238 and miR-1299.** (**A**) Transfection with miR-1238 and miR-1299 inhibitor led to decreased expression of miR-1238 and miR-1299 in circ_0005230-downregulated HuCCT1 cells. (**B**) Transfection with miR-1238 and miR-1299 mimics led to elevated expression of miR-1238 and miR-1299 in circ_0005230-upregulated KMBC cells. (**C, D**) Inhibition of miR-1238 and/or miR-1299 rescued the proliferation and invasion of circ_0005230-downregulated HuCCT1 cells. (**E, F**) Ectopic expression of miR-1238 and/or miR-1299 rescued the proliferation and invasion of circ_0005230-upregulated KMBC cells.*p<0.05, **p<0.01.

After confirming that circ_0005230 could interact with miR-1238 and miR-1299 in HuCCT1 and KMBC cells, it is imperative to evaluate whether the oncogenic activities of circ_0005230 were attributed to its modulation on miR-1238/1299. As Fig. 5A showed, HuCCT1 cells were cotransfected with si-circ_0005230 and indicated inhibitor miRNA to reverse elevated miR-1238 and miR-1299. In KMBC cell line, upregulation of circ_0005230 evidently attenuated miR-1238 and miR-1299 levels. Further cotransfected with miR-1238 or miR-1299 mimics remarkably restored its level (Fig. 5B). For the part of functional assays, si-circ transfected with either miR-1238 or miR-1299 inhibitor partly rescued the tumor suppressing functions caused by si-circ_0005230 in HuCCT1 cells. Furthermore, silencing of miR-1238 and miR-1299 at the same time almost totally reversed the tumor suppressing activities of si-circ_0005230 (Fig. 5C and D). In KMBC cells, cell growth and invasion potential were partly attenuated after cotransfected with indicated miRNA mimics. In the over-circ_0005230, miR-1238 and miR-1299 co-transfection group, cell growth and invasion capacities were further decreased (Fig. 5E and F).

### Circ_0005230 facilitates HuCCT1 cell growth and metastasis *in vivo*

**Figure 6:**
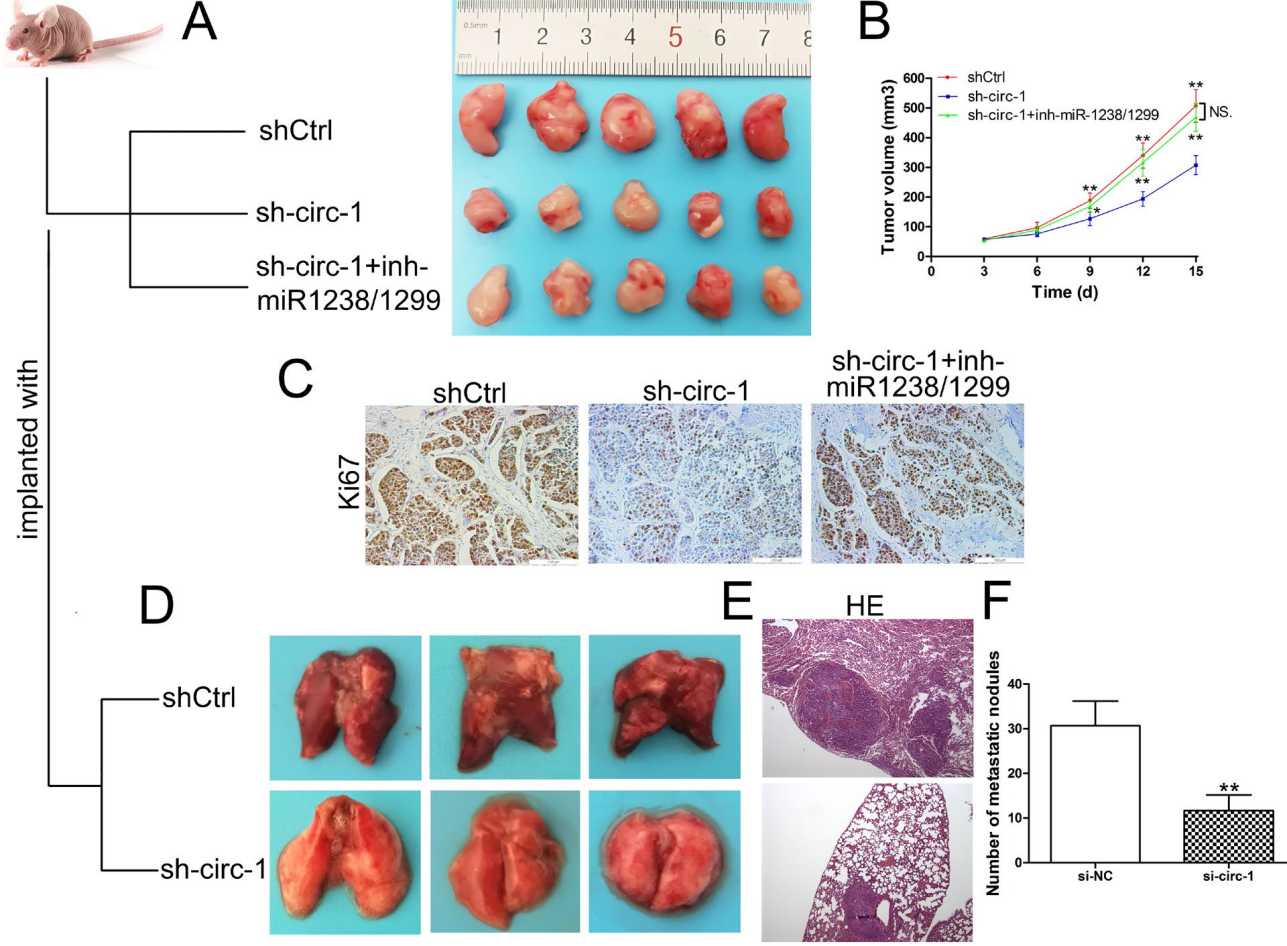
**Circ_0005230 promotes cell growth and metastasis *in vivo*.** (**A**) Tumors from nude mice after injection of transfected HuCCT1 cells. (**B**) Tumor volume was monitored every 3 days. (**C**) Ki67 expression and location were determined by IHC. (**D**) Lungs from experimental metastasis animal model of each group are shown. (**E**) HE staining was used to stain the resected lungs. (**F**) The number of tumor nodules on lung surfaces from two groups. *p<0.05, **p<0.01.

To validate the oncogenic role of circ_0005230 in CCA, *in vivo* study was performed. The xenografts formed from sh-circ_0005230-1 were significantly smaller in comparison with the shCtrl group tumors. In addition, sh-circ_0005230-1 cotransfected with miR-1238/1299 inhibitor could reverse the tumor suppressing role of sh-circ_0005230-1 (Fig. 6A and B). What’s more, the proliferative marker, Ki67 expression was weaker in the tumors formed from sh-circ_0005230-1 compared with control group. After co-silencing of circ_0005230, miR-1238 and miR-1299, Ki67 expression was elevated proved by IHC assay (Fig. 6C). Further lung metastatic tumor model demonstrated that downregulation of circ_0005230 resulted in less metastatic lung nodules, which is in accordance with the *in vitro* results (Fig. 6D-F).

## DISCUSSION

CCA is a devastating malignancy with increasing incidence across the world. Due to its insensitive to conventional chemotherapy, the outcomes of clinical therapy are dismal [1,2]. Emerging evidence verified that circRNAs can play critical roles in the pathogenesis of malignancies. For example, Li et al. revealed that upregulated hsa_circ_0007534 could regulate osteosarcoma cell progression by affecting AKT/GSK-3β signal [17]. Several studies also demonstrated the diagnostic or prognostic role of circRNAs in malignancies [18,19]. For CCA, we previous found Cdr1as might be regarded as a potential biomarker to predict tumor progression and poor prognosis [20]. In this study, the elevation of circ_0005230 was confirmed in CCA tissues specimens and cells in comparison with their matched non-cancerous samples and HIBEC, respectively. Moreover, the enhanced expression of circ_0005230 is related to larger tumor size (*p*=0.028), positive lymph node invasion (*p*=0.003), and higher TNM stages (*p*=0.020) for CCA patients.

Based on these observations, a series of functional assays were conducted on HuCCT1 (highest circ_0005230 expression) and KMBC cells (lowest circ_0005230 expression). The data documented that circ_0005230 could markedly enhance cell proliferation, migration and invasion potential and decrease cell apoptosis proved by CCK-8, clonogenic, flow cytmetric, AO/EB staining and transwell assays. Further animal study validated the *in vitro* data that circ_0005230 could facilitate cell growth and metastasis in CCA. These findings preliminary documented circ_0005230 as an oncogenic circRNA and a potential therapeutic target for CCA treatment. HIBEC cell line was used to explore whether knockdown of circ_0005230 may have side effect on normal biliary epithelial cells. The data indicated that silenced circ_0005230 did not affect the growth and apoptosis of HIBEC cells, which implied that the regulatory network of circ_0005230 in HIBEC is different from CCA cells and circ_0005230 does not play a major role in HIBEC cell growth. The gene expression regulated by circ_0005230 in HIBEC is different from CCA cells, indicating the mechanism of circ_0005230 is tissue specific.

CircRNAs could act as molecular sponges to bind to miRNAs or scaffold RBPs to exert their biological functions in carcinogenesis and tumor progression. For instance, Xiong et al. reported that circRNA ZNF609 upregulates FOXP4 expression to regulate renal carcinoma cell progression via sponging miR-138-5p [21]. Previously, we found increased expression of circ_0005230 could function as a competitive endogenous RNA to increase CBX8 expression by sponging miR-618 in BC [14]. Interestingly, miR-618 was almost not affected by circ_0005230 in the two selected CCA cells, which suggested that the mechanism of circ_0005230 maybe tissue specific. Among all the predicted miRNAs, only miR-1238 and miR-1299 were negatively correlated with circ_0005230 level. To clear whether circ_0005230 could directly interact with miR-1238/miR-1299, luciferase reporter assay was induced. In line with our expectation, the predicted binding sites were functional. The study relevant to the role of miR-1238 in cancer progression is rare. Only one report by Shi et al. identified miR-1238 as a tumor suppressor in cancer initiation and development. They uncovered that miR-1238 inhibits non-small cell lung cancer (NSCLC) cell growth partly by repressing LHX2 [22]. In the current study, we first proved miR-1238 as a tumor suppressive miRNA in CCA. MiR-1299 is a well-studied miRNA in some kind of malignancies and functions as a tumor suppressor. For example, Zhu et al. found miR-1299 could suppress hepatocellular carcinoma (HCC) cell growth by targeting CDK6 [23]. Another study indicated that ectopic expression of miR-1299 could downregulate STAT3 pathway to inhibit colon cancer cell growth [24]. Sang et al. demonstrated that Cdr1as maintains metastatic phenotypes of triple-negative breast cancer as a ceRNA of miR-1299 to target MMPs [25]. In this study, rescue experiments were used to uncover that the oncogenic role of circ_0005230 was partly attributed to its inhibition on miR-1238 and miR-1299. Previously, we have documented that Cdr1as is elevated in CCA, whether miR-1299 could also be sponged by Cdr1as in CCA is needed to further investigation. In addition, how miR-1238 and miR-1299 suppress CCA cell progression and the downstream targets of miR-1238 and miR-1299 are still needed to investigate. Along with future studies, circ_0005230 may be a rational therapeutic target for CCA.

Taken together, we identified circ_0005230 elevation in CCA tissues and cells and related to patients’ clinical severity. Furthermore, circ_0005230 plays an oncogenic role partly by sponging miR-1238 and miR-1299. Collectively, this work may help to form a novel effective therapeutics target for treating this devastating disease.

## MATERIALS AND METHODS

### Patient selection and tissue samples

The current study was reviewed by the Ethics Review Committees of the Second Affiliated Hospital of Harbin Medical University. Fifty-eight paired tissues and matched noncancerous specimens were acquired from the CCA patients who underwent operation from April 2012 to March 2014. Preoperative intervention was not adopted before the surgery and the samples were stored in liquid nitrogen.

### Cell culture

HCCC-9810 and RBE were purchused from Type Culture of Chinese Academy of Sciences (Shanghai, China). Normal biliary cell line, HIBEC, and the other CCA cells (KMBC, HuCCT1 and QBC939) were stored in our laboratory. Above five CCA cell lines and normal biliary epithelial cells were cultured in RPMI-1640 with 10% FBS (Invitrogen Life Technologies, USA) in a humidified air with 5% CO_2_ at 37°C.

### RNA isolation and qRT-PCR

Total RNA was isolated by Trizol (Thermo Fisher Scientific, USA). The Spectrophotometer (Implen, Germany) was applied to assess the concentration and quantity of the extracted RNA. Quantitative PCR was performed through Power SYBR Green (Takara Biotechnology, China). Relative expression of the genes were determined by 2^−ΔΔCt^.

### Cell transfection

Two siRNAs specifically targeting to the back-spliced site of circ_0005230 (si-circ-1/-2) and the negative control were acquired from GenePharma (Shanghai, China). The target sequences of si-circ_0005230 are shown below: si-circ-1: 5’-CAATATACTCTTTGTTTTGCA-3’. si-circ-2: 5’-CTCTTTGTTTTGCACACTAGG-3’. A pcDNA3.1 (+) circRNA mini vector was applied to clone the whole circRNA sequence and artificial inverted repeats. MiR-1238/1299 mimics/inhibitor were purchased from GenePharma (Shanghai, China). Lipofectamine 3000 was used for transfection according to the manufacturer’s protocols.

### Cell proliferation assays

Cell vitality was evaluated by CCK-8 (Beyotime, Beijing, China). The transfected cells in each 96-well plate were cultured overnight prior to adding with CCK-8 reagent in dark environment. OD values was quantified by a microplate reader at 450nm.

To detect the clone-forming ability of CCA cells, monoplast suspension of HuCCT1 and KMBC was seeded in 6-well plates at a same number of cells per well and incubated in medium containing 10% FBS. After 12 days, the macroscopic colonies were stained, photographed and calculated.

### Cell apoptosis detection

Apoptosis was measured by FACScan flow cytometer (BD Biosciences, USA). Transfected CCA cells were collected and incubated with 5μL of AnnexinV-FITC and 5μL of PI (BD, CA, USA) for 10min. The treated cells were subjected to flow cytometric analysis to detect apoptotic cells.

Acridine orange/ethidium bromide (AO/EB) double fluorescence staining assay was performed to further validate the data of flow cytometric analysis. The transfected HuCCT1 and KMBC cells were stained with prepared AO/EB mixing solution (1:1) for 5min before observation and photographing.

### Transwell assay

Cell metastatic properties were determined by Transwell experiments. Both migration and invasion assays were carried out through following protocols: serum-free cell suspensions were prepared and transferred to the upper chamber with 0.2ml cell suspension per well (pre-coated by Matrigel for invasion assay). The lower chamber was filled with medium supplemented with 10% FBS. Cells were cultivated for 24h and membranes were stained by crystal violet for 15min. At last, the cells were counted.

### Target prediction and luciferase reporter assay

Circular RNA Interactome was applied to predict the potential miRNAs targeting to circ_0005230. Dual luciferase reporter assay was carried out followed by the previous studies [14,26].

### Animal study

BALB/c nude mice (n=5 in each group) were purchased from Vital River (Beijing, China) and the study was authorized by the Animal Ethics Committee of the Second Affiliated Hospital of Harbin Medical University. Six weeks old female nude mice were received 8 × 10^6^ transfected HuCCT1 cells to induce xenograft. The volume of tumors were detected every three days. The mice were sacrificed at 15 days after injection. Immunohistochemistry (IHC) assay was conducted on the paraffin-embedded tissue samples from the mice to detect the location and relative expression of Ki67 (Abcam, Cambridge, MA, USA).

The stably transfected HuCCT1 cells (1 × 10^7^) were inoculated into the tail vein of each mice to construct the late stage lung metastatic model (n=3 in each group). Four weeks later, the mice were killed and their lungs were removed and subjected to hematoxylin and eosin (HE) dyeing. Finally, the number of lung metastases nodules were counted.

### Data analysis

All data are expressed as means ± standard deviation and analyzed using SPSS 19.0 software (SPSS, USA). The clinical significance of circ_0005230 in CCA was evaluated by using fisher’ exact test. Statistical difference was analyzed using *t*-test or one-way ANOVA. A *p* value less than 0.05 was considered statistically significant.
